# Genomic and Phenotypic Alterations of the Neuronal-Like Cells Derived from Human Embryonal Carcinoma Stem Cells (NT2) Caused by Exposure to Organophosphorus Compounds Paraoxon and Mipafox

**DOI:** 10.3390/ijms15010905

**Published:** 2014-01-09

**Authors:** David Pamies, Miguel A. Sogorb, Marco Fabbri, Laura Gribaldo, Angelo Collotta, Bibiana Scelfo, Eugenio Vilanova, Georgina Harris, Anna Bal-Price

**Affiliations:** 1 Bioengineering Institute, Miguel Hernández University, Elche 03202, Alicante, Spain; E-Mails: msogorb@umh.es (M.A.S.); evilanova@umh.es (E.V.); 2 Institute for Health and Consumer Protection, European Commission Joint Research Centre, Ispra, Varese 21027, Italy; E-Mails: marco.fabbri@gmail.com (M.F.); laura.gribaldo@ec.europa.eu (L.G.); angelo.collotta@jrc.ec.europa.eu (A.C.); bibiana.scelfo@gmail.com (B.S.); georgina.hlopez@gmail.com (G.H.); anna.price@jrc.ec.europa.eu (A.B.-P.); 3 Bloomberg School of Public Health, Johns Hopkins University, CAAT, Baltimore, MD 21205, USA; 4 Department of Experimental and Clinical Medicine, University of Insubria, Varese 21100, Italy

**Keywords:** neurodifferentiation, neurodevelopmental toxicity, NT2, organophosphorus pesticides, NTE, human embryonal carcinoma stem cells

## Abstract

Historically, only few chemicals have been identified as neurodevelopmental toxicants, however, concern remains, and has recently increased, based upon the association between chemical exposures and increased developmental disorders. Diminution in motor speed and latency has been reported in preschool children from agricultural communities. Organophosphorus compounds (OPs) are pesticides due to their acute insecticidal effects mediated by the inhibition of acetylcholinesterase, although other esterases as neuropathy target esterase (NTE) can also be inhibited. Other neurological and neurodevelopmental toxic effects with unknown targets have been reported after chronic exposure to OPs *in vivo*. We studied the initial stages of retinoic acid acid-triggered differentiation of pluripotent cells towards neural progenitors derived from human embryonal carcinoma stem cells to determine if neuropathic OP, mipafox, and non-neuropathic OP, paraoxon, are able to alter differentiation of neural precursor cells *in vitro*. Exposure to 1 μM paraoxon (non-cytotoxic concentrations) altered the expression of different genes involved in signaling pathways related to chromatin assembly and nucleosome integrity. Conversely, exposure to 5 μM mipafox, a known inhibitor of NTE activity, showed no significant changes on gene expression. We conclude that 1 μM paraoxon could affect the initial stage of *in vitro* neurodifferentiation possibly due to a teratogenic effect, while the absence of transcriptional alterations by mipafox exposure did not allow us to conclude a possible effect on neurodifferentiation pathways at the tested concentration.

## 1. Introduction

Organophosphorus compounds (OPs) have been used worldwide as pesticides for insect control. In Europe alone, OP insecticide production was close to 31,000 tons in 2010 [1]. The American Association of Poison Control Centers reported almost 4000 exposures to OPs and four exposure-related fatalities in 2010 [2]. Many OPs target the nervous system of insect pests. Because of the similarity of neurochemical processes, these compounds are also likely to be neurotoxic to human brain, especially to the developing nervous system which is inherently much more vulnerable to injury caused by toxic agents than the brain of adults [3–5]. Indeed, there are evidences that pesticides reduce motor activity, latency and cause visuospatial deficits after high exposure [6,7]. The main mechanism of action of OPs is based on irreversible enzyme inactivation caused by the phosphorylation of the active centre of esterases located in the neurons of the central and peripheral nervous systems [8]. The inhibition of esterases can cause two main types of poisoning effects. The insecticidal effects of OPs are specifically due to acetylcholinesterase (AChE) inhibition, which is essential for the transmission of nervous signals in target (insects) and non-target (e.g., humans and other mammals) species [8]. However, another syndrome has been described after exposure to some OPs, the so-called organophosphorus-induced delayed polyneuropathy. This is a paralyzing syndrome characterized by the degeneration of nerve axons, which is clinically detectable 14 to 16 days post-exposure [9]. Conversely, this delayed neuropathy is triggered by the phosphorylation and further chemical modification of an esterase (other than AChE) called Neuropathy Target Esterase (NTE) [9].

In addition, low-level chronic exposures, which usually take place in occupational environments, can cause other neurotoxic effects. Incidences of neurological and neurodegenerative diseases have been reported in epidemiologic studies performed with pesticide spreaders, greenhouse workers, agricultural workers and farmers occupationally exposed to pesticides (including OPs) [10–12]. OPs also might be developmental toxicants due to the inhibition of esterases during pregnancy, oxidative stress or endocrine disruption [13]. It has also been suggested that the neurodevelopmental toxicity of OPs may be due to direct interference with the morphogenic activity that AChE normally displays during neurodevelopment [14,15], which might alter neural connectivity, induce long-lasting changes in spatial learning and memory formation [16], or alter the expression of genes involved in nervous system development [17,18], as it is specifically described for the OP chlorpyrifos. Epidemiologic and laboratory animal studies suggest that pesticides (OPs, carbamates, pyrethroids and others) can cause developmental neurotoxicity [3]. In addition, an anthropological study of children aged 4–5 years in Mexico showed that children highly-exposed to pesticides demonstrated decreases in stamina, gross and fine eye-hand coordination, perturbation in short term memory, and the ability to draw a picture of a person [19].

Neurotoxic effects depend on the developmental window in which the exposure takes place and can be particularly severe in early stages, when complex cellular-molecular processes of neural progenitor cell commitment, cell proliferation, migration and differentiation take place [4,5,20]. Damage to any of these processes can cause a variety of adverse health effects, such as mental retardation, altered behavior and other neurodevelopmental diseases [21].

Human-derived NTera2/D1 (NT2) cells have the capacity to differentiate into fully mature neuronal and astrocyte-like cells [22]. During this process, the neuron-like cells derived from NT2 neural progenitor cells could serve as a valuable model for developmental neurotoxicity studies since various cell differentiation stages can be followed in these cultures [23,24].

The main goal of this study is to determine whether exposure to model OPs during retinoic acid (RA)-induced differentiation of pluripotent cells is able to affect the neural commitment of progenitor cells followed by the alterations in the initial process of neuronal differentiation. Indeed, this very early exposure of neural progenitor cells to environmental toxicants seems to be one of the most sensitive time intervals during development causing neurotoxicity [25,26]. Microarray technology has been used to screen the developmental neurotoxic effects induced by OPs [27]. We studied alterations in the expression of the whole human genome in RA-induced differentiation of pluripotent cells towards neural progenitors from NT2 cells by exposure to non-neuropathic OP paraoxon (the physiologically active derivative of the insecticide parathion) or neuropathic OP mipafox. Non-cytotoxic concentrations of paraoxon affected the expression of several genes related mainly to DNA and nucleosome integrity. In contrast, mipafox exposure showed no changes in gene expression.

## 2. Results

### 2.1. Cell Viability after Exposure to OPs

**Figure 1.**
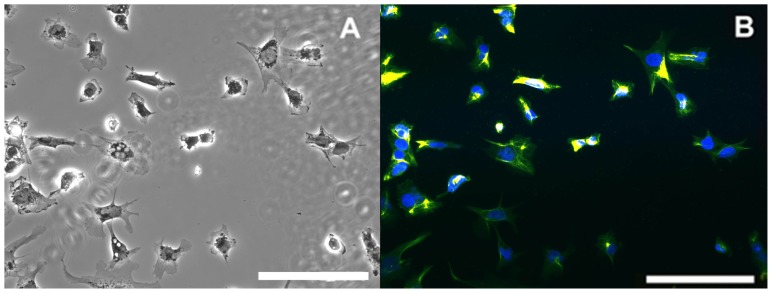
NT2 neural progenitor cells. (**A**) Phase contrast images showing NT2 neural progenitor cells; (**B**) Expression of NPC (neural progenitor cells) marker, nestin, co-stained with 4′,6-diamidino-2-phenylindole (DAPI) show 100% positive nestin cells. Bars correspond to 500 μm.

**Figure 2.**
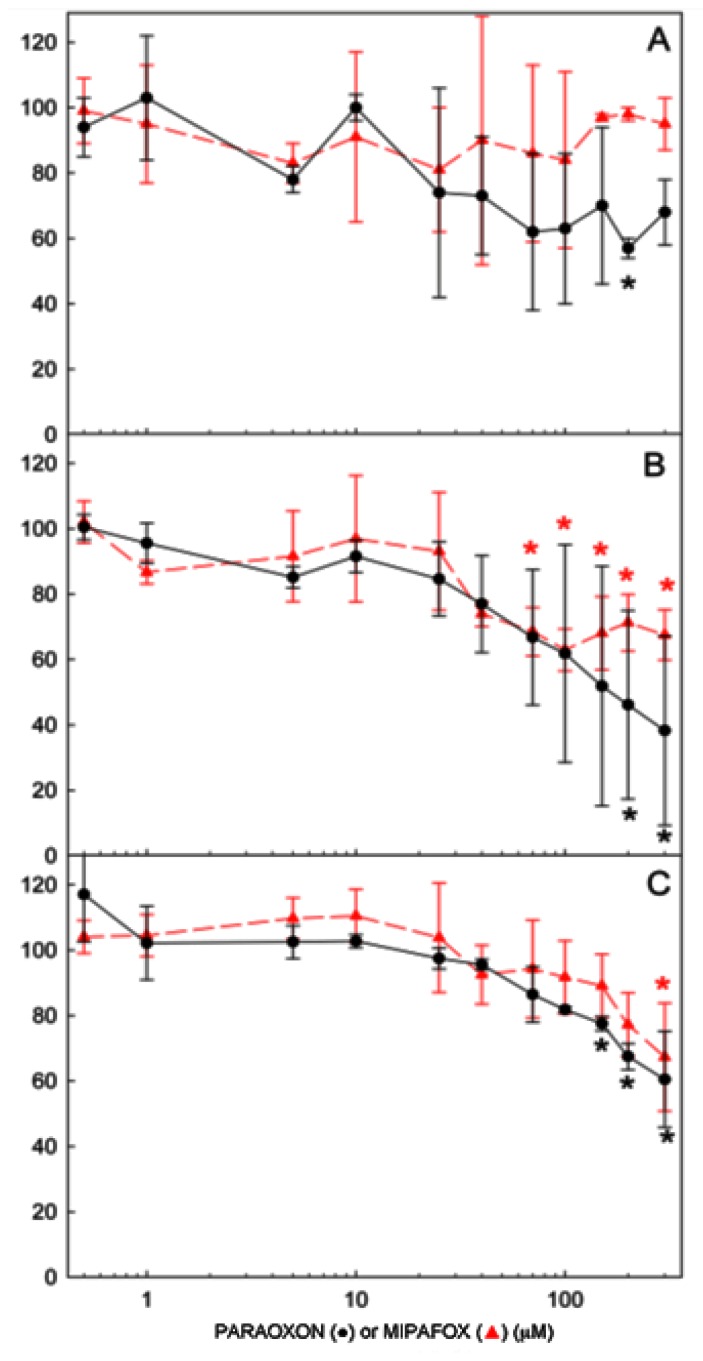
Effect of paraoxon and mipafox on cell viability of NT2 cells during the first stage of neurodifferentiation measured by MTT assay. Cells were exposed to 0.5, 1, 5, 10, 25, 40, 70, 100, 150, 200 and 300 μM of either paraoxon (●) or mipafox ( 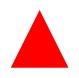) for 4 days (**A**); 10 days (**B**); and 15 days (**C**). Data represent mean ± SEM of the four independent experiments with 16 independent technical replicates for each experimental condition run. (* statistically different from the controls for *p* < 0.05 in both paraoxon and mipafox by Dunnett’s test).

In the dose response assessment of paraoxon and mipafox, NT2 cells were exposed for 4, 10 and 15 days to several paraoxon and mipafox concentrations ranging between 0.5 and 300 μM. After 4 days exposure to paraoxon, only concentrations higher than 200 μM reduced cell viability (*p* < 0.05) (Figure 1), while none of the tested mipafox concentrations had any effect as evaluated by MTT assay. However, after 10 days of mipafox exposure to the concentrations higher than 70 μM, the cells showed significant loss in cell viability (Figure 1). After longer time of exposure, 15 days, paraoxon and mipafox significantly reduced cell viability (*p* < 0.05) at concentrations higher than 100 μM and 200 μM respectively, while 1 μM paraoxon and 5 μM mipafox did not alter viability (Figure 2). Based on these results, 1 μM paraoxon and 5 μM mipafox were selected for transcriptomics studies as non-cytotoxic concentrations.

### 2.2. Effect of Paraoxon and Mipafox on NTE Activity

**Figure 3.**
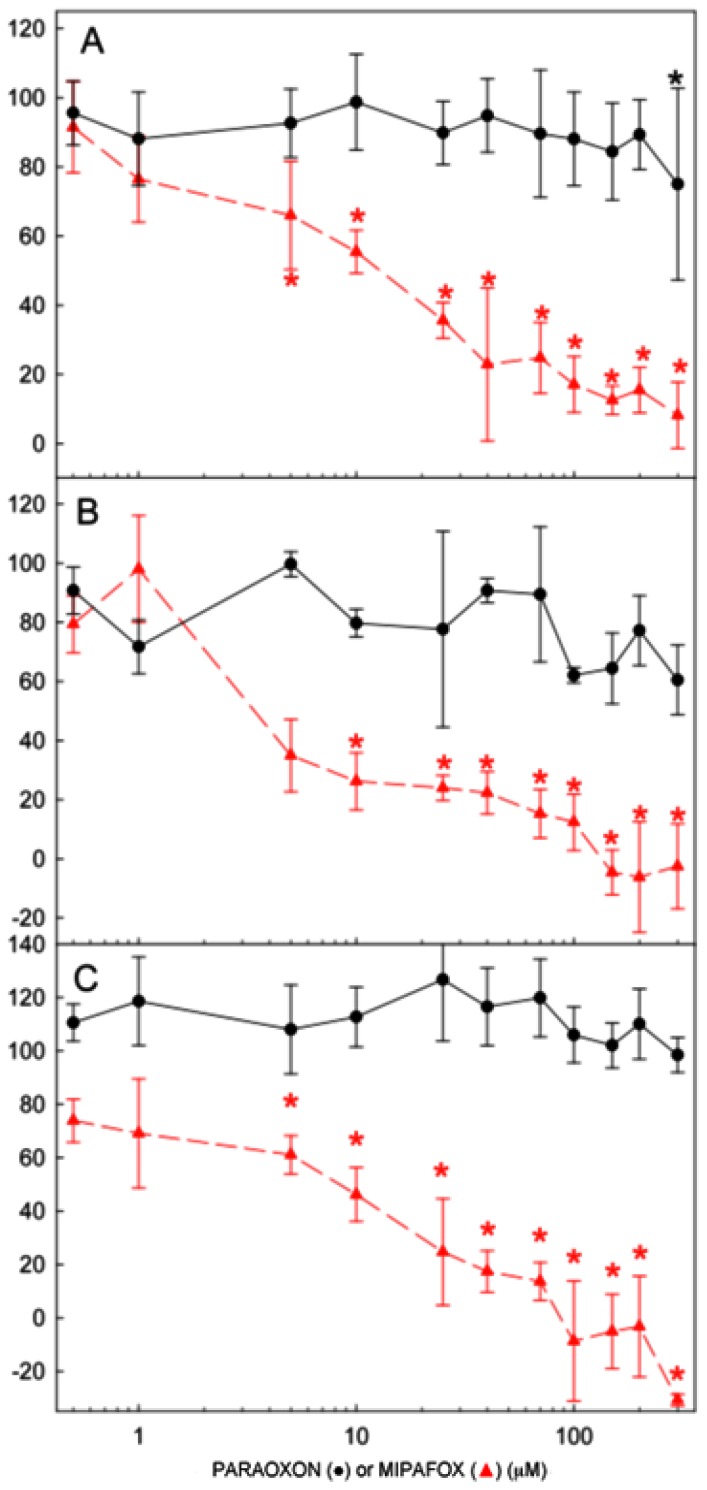
Changes in NTE activity of the NT2 cells exposed to paraoxon or mipafox during the neurodifferentiation process. Cells were exposed to 0.5, 1, 5, 10, 25, 40, 70, 100, 150, 200 and 300 μM of either paraoxon (●) or mipafox ( 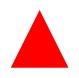) for 4 days (**A**); 10 days (**B**); and 15 days (**C**). NTE activity is expressed as the percentage of activity determined in the time-matched control (non-exposed) cultures. Data represent mean ± SEM of three independent experiments with 8 technical replicates for each experimental condition. (* statistically different from the controls for *p* < 0.05 with a Dunnett’s test).

Non-neuropathic OP paraoxon did not inhibit NTE after 4, 10 or 15 days of exposure (Figure 3). Conversely, neuropathic OP mipafox caused an extensive inhibition of NTE (Figure 3). This inhibition was significant after 4 days of exposure to 5 μM mipafox, and reached approximately 8% of control activity after exposure to 300 μM (Figure 3). Similar results were observed after 10 and 15 days of exposure.

### 2.3. Microarray Analysis after 4-Day Exposure

**Figure 4.**
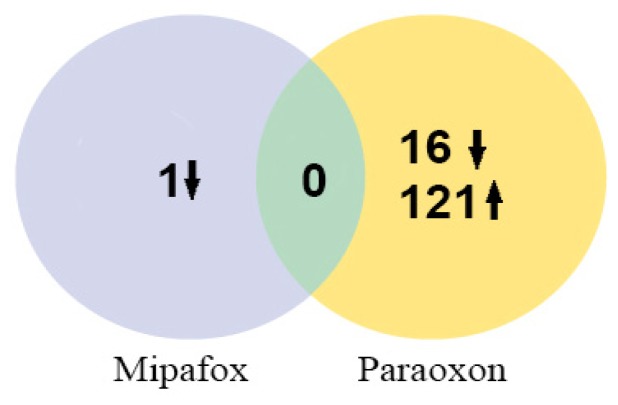
Venn diagram of the genes with altered expressions after exposure to paraoxon and mipafox. Cells were exposed to 1 μM paraoxon or 5 μM mipafox for 4 days. Afterwards, the whole human genome expression was recorded using microarrays, as described in Section 4.6. Each compartment represents the number of genes with a statistically (at least corrected *p* < 0.05) altered expression found in each condition. ↓ down-regulated genes, ↑ up-regulated genes.

The mRNA expression across the whole human genome was evaluated in NT2 cells during the initial stage of RA-induced differentiation of pluripotent cells towards the neural committed progenitor cells after 4 days of exposure to 1 μM paraoxon or 5 μM mipafox (both are non-cytotoxic concentrations) using microarray analysis. Paraoxon caused a statistically significant alteration in the expression of 137 genes, while exposure to mipafox altered the expression of a single gene (Figure 4). No overlapping was noted between the genes altered by paraoxon exposure and the single gene altered by mipafox exposure (Figure 4). The one gene modified by mipafox treatment was a long non-coding RNA, a non-protein coding transcript related with a transcription function.

The data obtained from gene expression studies was further analyzed with the DAVID software using the Gene Ontology database separated into three parts: biological process, molecular function and cellular components [28]. For analysis purposes, only those genes with a fold change higher than 2 or lower than 0.5 and with a corrected *p*-value lower than 0.05 were considered. The highest enrichment score was found for a cluster of genes related to nucleosome and chromatin assembly (Table 1). An independent analysis of the up-regulated and down-regulated genes showed that these modifications were attributable to down-regulated genes (raw data in Supplementary Materials 2). Table 2 shows the genes which mRNA levels altered up-regulated and down-regulated after paraoxon exposure. The function of most of these genes is related to chromatin assembly and chromatin regulation (HIST1H2AB, HIST1H4E, CBX3), apoptosis (FKSG2 and UBE2Z) or cell–cell signaling and differentiation (FGFR1, YAP1, SRGAP2P2).

### 2.4. Effect of Paraoxon and Mipafox on the Morphology of NT2-Derived Neurons

**Figure 5.**
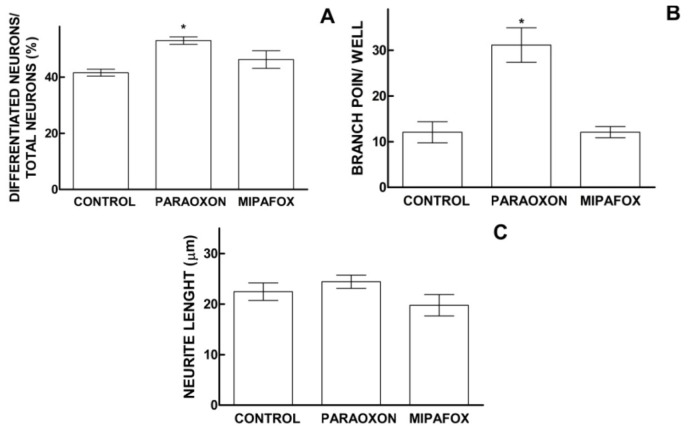

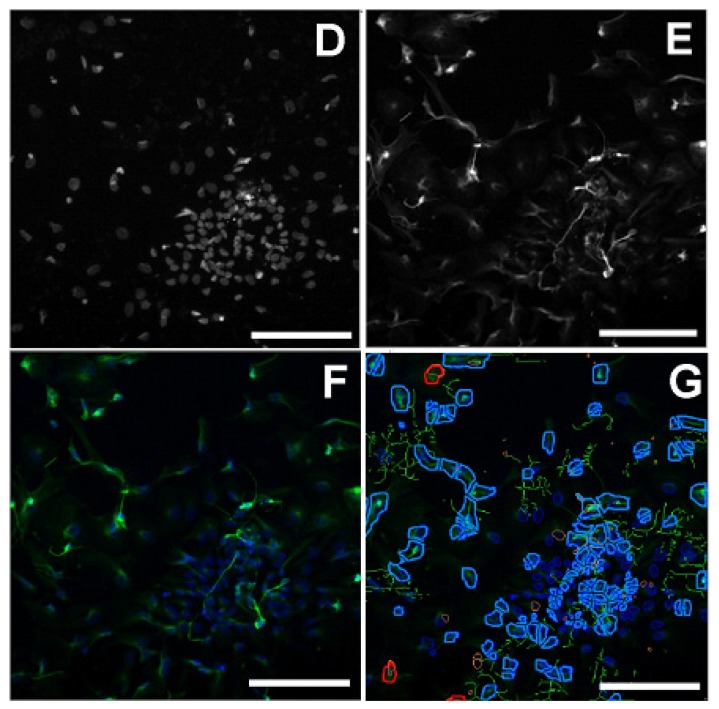
Effects of paraoxon and mipafox on the neural differentiation. During the initial process of neural differentiation NT2 cells were seeded on 96-well plates and exposed to 1 μM paraoxon or 5 μM mipafox for 13 days. Using the Cellomics ArrayScan device the following morphological parameters of the cells positively stained against β-Tubulin III were quantified: differentiating neuronal-like cells (cells with more than 3 neurites or with a neurite total length longer than 6.5 μm) (**A**); number of branch points (**B**) and length of neurites (**C**); Each Figure shows the results obtained together with a representative picture, randomly selected among those employed for quantification. Each experimental condition was assayed in 16 wells (6 different pictures per well). Plots represent mean ± SEM of all records performed per each experimental condition. * statistically different from the controls for *p* < 0.05 with Dunnett’s test. Pictures show morphology and staining of analyzed cultures (white bars represent 500 μm); (**D**) Nuclear staining with DAPI; (**E**) Staining of neuronal bodies with â-tubulin III; (**F**) Double staining (green for β-tubulin III and blue for DAPI); and (**G**) The same picture analyzed Cellomics ArrayScan device. Note in (**G**) blue cellular outlines for neurons with neurites meeting the above defined threshold, while red outlines remark neuronal bodies that were not quantified because they did not meet the set threshold. See also in (**G**) neurites (green) and nuclei (blue) that were not outlined for quantification because they were not considered as neurons.

The morphology of NT2 cells differentiating towards neuronal-like phenotype for 13 days (in the presence of RA) were stained positively against β-Tubulin III (neuronal specific marker) and their morphology was analyzed using the imaging platform Cellomics ArrayScan vTi (Thermo Scientific Cellomics^®^, Pittsburgh, PA, USA), as described in Section 4.6. Paraoxon caused a statistically significant increase (11.4% regarding the control, *p* < 0.05) in the total number of differentiated neuronal-like cells (cell bodies with more than 3 processes or with processes whose total length was longer than 6.5 μm) present in cultures, while no differences in this parameter were recorded between the control and mipafox-exposed cultures (Figure 5A). Likewise, the total number of branch points in differentiating cells was higher (258% of control, *p* < 0.05) in the cultures that were differentiating in the presence of paraoxon than in the control cultures, while the presence of mipafox did not alter the number of branch points (Figure 5B). However, the cultures exposed to both paraoxon or mipafox displayed no significant differences in terms of total length of neurites per well (Figure 5C) and total number of dense (non-apoptotic) nuclei per well (data not shown).

## 3. Discussion

The main mechanism for acute toxic effects of OPs is the inhibition of AChE in the nervous system. Nevertheless, other deleterious effects have been reported for these pesticides, such as inhibition of other esterases (*i.e.*, NTE), damage to DNA and RNA synthesis [29], dysregulation of signal transduction pathways [30], oxidative stress [30], astroglial proliferation [31], embryogenesis dysfunction [32,33].

In this work, we used a neuronal progenitor cells of undifferentiated NT2 cells in the presence of RA, to study the effects of mipafox (a neuropathic OP capable of inhibiting NTE and inducer of the so-called organophosphorus induced polyneuropathy) [34] and paraoxon (a non-neuropathic OP that is not able to inhibit NTE) [35,36] on the initial stages of neuronal differentiation process under *in vitro* conditions. This early exposure of neural progenitor cells to environmental chemicals seems to be one of the most sensitive time intervals of exposure in the context of developmental neurotoxicity. Indeed, it has been shown that NT2 derived neural precursors were already affected by non-cytotoxic concentrations of methyl mercury [25,37]. In contrast these concentrations did not affect the markers of more mature cells, indicating that the early window of exposure is more sensitive than later stages of neuronal differentiation. A similar conclusion was obtained based on the exposure of a human neural precursor cells derived from umbilical cord blood (HUCB-NSC) to various developmental neurotoxic compounds, including methyl mercury chloride. Less differentiated cells of HUCB-NSC were more sensitive to neurotoxicants [25] than the cells at the later stage of neuronal differentiation. Compounds that can affect the very initial stage of neural precursor’s commitment are potential developmental neurotoxicants and this early exposure can be critical for changes observed in the neuronal/glial ratio and/or neuronal/glial cell morphology and function. NT2 are capable to differentiate in both neuronal and glial cells.

In this study mRNA levels have been found to be modified based on microarray analysis of the cells exposed to paraoxon during 4 days. Preliminary studies have shown that after 13 days of exposure to paraoxon, the cells differentiating towards neuronal phenotype (stained positively against β-Tubulin III) have a tendency towards increased number of branch points and higher number of differentiated neuronal-like cells (Figure 5). However, further studies are needed to confirm this. The results obtained from mRNA expression modification point towards the fact that paraoxon could have an effect on early stages of neural precursor commitment and the initial differentiation into neuronal-like cells.

### 3.1. Effects of Mipafox during NT2 Neurodifferentiation

Previous studies with mipafox have reported that different NTE inhibiting neuropathic OPs caused reduced neurites length in the SH-SY5Y human neuroblastoma [38], N2a mouse neuroblastoma [39], C6 rat glioma [40], sympathetic neurons deriving from superior cervical ganglia [41], chick embryo dorsal root ganglia [42] or PC12 cells [43]. Moreover, Henschler *et al.* [44] reported that 26 different neuropathic OPs reduced the length of the neurites-like processes induced by dibutyryl cAMP in the N18 mouse brain neuroblastoma and processes of the C6 rat brain glioma cells while eight non-neuropathic OPs did not. However, the studies in which EB2.2 mouse embryonic stem cells with the knocked-out NTE encoding gene were used; a delay in the onset of neurite outgrowth (but no effect on their total length) was observed [45]. The above-reported differences can be explained on the basis of different cellular models, different concentrations applied, time of exposure and on mechanisms of reduction of NTE activity (chemical inhibition of the protein with mipafox or other neuopathic OPs in SH-SY5Y, C6, PC12, N18 cells and chick embryo dorsal root ganglia or genetic silencing in EB2.2 cells and D3 mouse embryonic stem cells). NT2 is a human embryonal carcinoma cell line classified as stem cells that can differentiate into post-mitotic neurons under RA exposure [22], while the other cellular systems used for studying alterations in neurites outgrowth are either somatic stem cells or adult cells. Therefore, it can be expected that these cells were at different stages of differentiation processes during mipafox exposure, which might determine the response of differentiating cells to the insult of the OP.

In our studies no genetic modifications due to mipafox exposure have been reported. However, interference RNA for Pnpla6 (NTE encoding gene) has shown different genetic modifications in D3 mouse embryonic stem cells during the differentiation of embryonic bodies [32]. A reduction of NTE enzymatic activity in NT2 cells did not produce any modifications in gene expression. Again, these differences could be due to the different cellular models used, different concentrations applied and time of exposure as well as different mechanisms of reduction of NTE activity (genetic repression of Pnpla6 or NTE enzymatic activity inhibition).

### 3.2. Effects of Paraoxon during Initial Stage of NT2 Neurodifferentiation

Parathion and, in consequence, the physiologically active derivative paraoxon have shown to induce developmental neurotoxicity effects in humans [46]. Paraoxon exposure caused changes in mRNA levels as determined by microarray analysis already after 4 days of neuronal differentiation triggered by RA. These findings may support our preliminary results where increased neuronal differentiation (number of total differentiating cells towards neuronal phenotype and number of branch points (Figure 5) after exposure to paraoxon was observed. Such effect could change the networking between the mature neurons that can be obtained in this cell model. Indeed, based on our early studies [47] these cells have capacity to differentiate into fully mature neurons following the protocol described by Pleasure [48].

The pathway analysis performed with the DAVID bioinformatics tool for the 137 genes with altered expression strongly suggests an association with the pathways related to DNA integrity and to nucleosome and chromatin assembly (Table 1). The analysis of the altered mRNA levels of different genes (Table 2) reveals that they belong to different families, such as CBX3, related to chromatin regulation [49], or HIST1H4E and HIST1H2AB, related with nucleosome assembly [50], or YAP1, related with the regulation of different processes in development [51]. These results are consistent with other reports where DNA damage in the lymphocytes of workers occupationally exposed to OPs was detected [52–54].

The exposure to paraoxon down-regulates also the mRNA levels of growth factor receptor 1 (FGFR1) that is involved in cellular proliferation. It was demonstrated, for example, that FGFR1-deficient mice develop an abnormal olfactory bulb due to failure in the decrease of cell proliferation, indicating that FGF signaling is required to inhibit proliferation at the anterior tip of the forebrain [55]. Yes-associated protein 1 (YAP1) is a transcriptional co-activator that controls cell proliferation and differentiation in a variety of tissues during development. An increase in differentiation in mouse retina cells after silencing has been demonstrated [56]. Both mRNA levels of (FGFR1 and YAP1) were down-regulated after 4 days of exposure to paraoxon (Table 2), which could support the increase in the number of differentiated neurons found 13 days after neurodifferentiation (Figure 5). Additionally, mRNA levels of other genes such as *FGFR1*, *SGSM2*, *SET*, *SCD5* related to nervous system development have been modified. However, paraoxon and paraoxon-methyl have been also shown to produce teratogenicity and genotoxicity in different models [57–59]. Moreover, gene set enrichment analysis has shown mRNA levels modify of genes related with apoptosis and cell size regulation (Table 1) function. Taking these results into consideration, it is possible that the effects observed in our study may be due to teratogenicity induced by paraoxon. Indeed, microarrays analysis have shown impaired chromosome packaging and organization (Table 1) that could produce chromosome aberration, normally linked with teratogenicity effects.

## 4. Material and Methods

### 4.1. Chemicals

Paraoxon (O,O′-diethyl *p*-nitrophenyl phosphate) was obtained from Sigma-Aldrich S.A. (Madrid, Spain) and mipafox (*N*,*N*-diisopropyl diamidophosphorofluoridate) was purchased from Lark Enterprise (Webster, MA, USA).

### 4.2. NT2 Cell Differentiation into Neuronal-like Cells

The NTERA-2 cl. D1 (NT2) cell line derived from human teratocarcinoma, was purchased from the American Type Culture Collection (Rockville, MD, USA). Two different culture media (for proliferation of neural progenitor cells (NPC) and for neuronal differentiation) were used according to the protocol described by Pleasure *et al*. [48]. Initially, the neural progenitor NT2 cells (Figure 1) were cultured in uncoated 75-cm^2^ flasks (Nunc, New York, NY, USA) at a density of 4 × 10^4^ cells/cm^2^ and maintained in Opti-MEM (Gibco) media supplemented with 5% heat-inactivated FBS (HyClone, Logan, UT, USA), plus 50 U penicillin/mL and 100 μg/mL streptomycin (Gibco, Carlsbad, CA, USA). In order to induce neural differentiation, cells were trypsinized and cultured in DMEM-HG (Gibco) medium supplemented with 10 μM retinoic acid (RA; Sigma, St. Louis, MO, USA), 10% FBS (HyClone, Logan, UT, USA ), 50 U penicillin/mL and 100 μg/mL streptomycin. During the RA-induced differentiation process, the NT2 cells were seeded in different plate formats (see following sections), depending on the experimental aim, and exposed to OPs (paraoxon and mipafox). OPs were freshly dissolved in cell culture media which was replaced every 2 days.

### 4.3. Assessment of Cell Viability Using the MTT Assay

To select the non-cytotoxic concentrations of paraoxon an mipafox MTT assay was performed that is widely used for evaluation of the *in vitro* cell viability in toxicity studies [20,28].

Cells were seeded at a density of 25 × 10^3^ cells/cm^2^ in uncoated 96-well plates (BD, Franklin Lakes, NJ, USA) and cultured in RA-induced differentiation media being simultaneously exposed to different paraoxon and mipafox concentrations ranging between 0 and 300 μM (0, 0.5, 1, 5, 10, 25, 40, 70, 100, 150, 200 and 300 μM) for 4, 10 and 15 days starting treatment on day 0 of differentiation. Cell culture medium was changed every 2 days. Cells were washed twice with phosphate buffered saline (PBS) (137 mM NaCl; 2.7 mM KCl, 8.1 mM Na_2_HPO_4_, 1.5 mM KH_2_PO_4_) and 200 μL of 3-(4,5-dimethylthiazol-2-yl)-2,5-diphenyltetrazolium bromide (MTT) solution (1 mg/mL) were added to each well. After 3 h the cells were washed with PBS and 100 μL of dimethyl sulfoxide were added to each well. Plates were submitted to shaking (50 rpm, 10 min) to ensure a complete dissolution of formazan crystals. Finally, absorbance was read at 540 nm in a microplate reader (Infinite^®^ 200 PRO series, Tecan Group Ltd., Männedorf, Switzerland) and the percentage of cell viability after exposure was calculated by assuming 100% of viability for the absorbance recorded in the control (non-exposed) cultures. Four independent experiments per time point were performed in each of which sixteen independent technical replicates (independent wells) were used to test each experimental condition (Figure 2).

### 4.4. NTE Enzymatic Activity

NTE activity was determined by the method of Pamies *et al.* [30] that is defined as phenyl valerate (PV) esterase (PVase) activity resistant to paraoxon and sensitive to mipafox. B (paraoxon-resistant) and C activity (resistant to both paraoxon and mipafox) are discriminated by the result from the differential sensitivities of NT2 PV esterases to nonneurotoxic paraoxon and to neurotoxic mipafox [36]. Briefly, B activity was defined and recorded as the PV hydrolyzing activity in those samples preincubated for 30 min at 37 °C with 40 μM paraoxon. C activity was defined and recorded as PV hydrolysing activity in the samples preincubated with 40 μM paraoxon plus 250 μM mipafox. In this method, NTE activity is calculated as B-C. Three independent experiments were performed in which 8 independent technical replicates were done to test each experimental condition (B and C activity measurements) at three time points (4, 10 and 15 days).

Cells cultured under RA-induced neurodifferentiation were seeded at density of 2 × 10^5^ cells/cm^2^ in 96-well uncoated plates. These cells were exposed during 4, 10 and 15 days to OPs at the concentrations ranging between 0 and 300 μM (0, 0.5, 1, 5, 10, 25, 40, 70, 100, 150, 200 and 300 μM), starting the exposure at day 0 of differentiation. On each measurement day, cells were washed with PBS and incubated with 40 μM paraoxon alone or in combination with 250 μM mipafox in PBS for 30 min at 37 °C to record B and C activities. The medium containing paraoxon or mipafox was removed and 100 μL of 7.5 mM PV in PBS were added to each well and incubated at 37 °C for 60 min. The reaction was stopped by adding 100 μL of 2% SDS-0.25 mg 4-aminoantipyrine/mL in 50 mM TRIS-1 mM EDTA buffer (pH 8.0). After 15 min at room temperature, 50 μL of 1% (*w*/*v*) potassium ferricyanide (in water) were added. The released phenol was quantified by recording absorbance at 510 nm and by comparing to the standard curve of the phenol. NTE enzymatic activity (B-C) across three independent experiments was determined and plotted against time (Figure 3). In addition, NTE enzymatic activity was measured in parallel to microarray analysis experiments (Figure S1).

### 4.5. Microarray Studies

Maximum inhibition of NTE appeared 2 days after exposure to mipafox (Figure S1). For this reason, four days exposure to paraoxon and mipafox during RA-induced differentiation were chosen for the microarray study as this allowed two additional days for any alterations caused by NTE inhibition to translate to all complex genetic pathways. The concentrations tested were based on the following rational; 1 μM paraoxon is high enough to inhibit AChE and other esterases not related with NTE after chronic exposures such as those employed in this study, because these enzymes are usually inhibited after 30 min exposure to paraoxon concentrations ranging between the nM and μM units [35,60–62]. Similarly, 5 μM mipafox is able to cause significant inhibition of NTE, as we confirmed in experiments described in Section 4.4. It has been shown that this concentration also inhibits AChE since the ratio between *IC*_50_ for inhibition of AChE and NTE by mipafox ranges between 1 and 10 [60].

For microarray studies the cells were cultured in petri dishes (60 mm, at the initial cellular density of 5 × 10^4^ cells/cm^2^) under RA-induced differentiation conditions (as described in Section 4.2.) for 4 days in the presence of 1 μM paraoxon, or 5 μM mipafox. After 4 days of the exposure to OPs RNA was isolated from treated cells and the control culture (non-treated). In parallel with microarray gene expression studies, the NTE enzymatic activity experiment was performed (Figure S1).

The RNeasy Plus kit (Qiagen, Germantown, MD, USA) was used. Total RNA was quantified using a ND-1000 UV-Vis Spectrophotometer (NanoDrop Technologies, Wilmington, DE, USA) and its integrity was assessed with the Agilent 2100 Bioanalyzer (Agilent, Milano, Italy) according to the manufacturer’s instructions. All the RNA samples used in this study met the criteria of 260/280 ratio above 1.9 and an RNA Integrity number above 9.0. The microarray experiment was designed to perform three technical replicates for each treatment and all the samples were isolated and process at the same time. Sample-labelling, hybridization, washing and scanning steps were conducted following the manufacturer’s specifications. In short, Cy3-labelled cDNA was generated from 500 ng of input total RNA using the Quick Amp Labeling Kit, One-color (Agilent). For each sample, 1.65 μg of cDNA from each labelling reaction (with a specific activity above 9.0) was hybridized using the Gene expression Hybridization Kit (Agilent) to the Agilent Whole Human Genome Oligo Microarray (Agilent), which is a 4 × 44 k 60 mer slide format where all 4 arrays represent about 41,000 unique genes and transcripts. After hybridization, slides were washed and then scanned in the Agilent G2565BA Microarray Scanner. The fluorescence intensities on the scanned images were extracted and pre-processed by the Agilent Feature Extraction Software (version 10.5.1.1, Agilent, Milan, Italy). Quality control and array normalization were performed in the R statistical environment using the Agi4 × 44PreProcess package downloaded from the Bioconductor web site. The normalization and filtering steps were based on those described in the Agi4 × 44PreProcess reference manual. In order to detect expression differences among different cell populations, a moderated *t* test was applied. Moderated t statistics were generated by the Limma Bioconductor package. Differentially expressed genes were defined as those with a log (base 2) fold change higher than 1 or lower than −1, and a false discovery rate (Benjamini and Hochberg’s method) corrected by *p*-value smaller than 0.05 [63]. All the above computations were conducted using the R statistics programming environment.

The expression microarray data have been deposited in the NCBI Gene Expression Omnibus and are accessible through GEO series accession number GSE38050 (NCBI. Available online: http://www.ncbi.nlm.nih.gov/geo/query/acc.cgi?acc=GSE38050 (accessed on 26 December 2013)).

The microarray results were analyzed by the DAVID (Nature. Available online: http://www.nature.com/nprot/journal/v4/n1/abs/nprot.2008.211.html, accessed on 26 December 2013) database. The analysis identified the biological functions that were altered by exposure to OPs. A right-tailed Fisher’s exact test was used to calculate a *p*-value to determine the probability that each biological function assigned to that data set is due to chance alone.

### 4.6. Evaluation of Cell Morphology Using Fluorescence Imaging

RA-induced cell differentiation was performed at density of 4160 cells/cm^2^ in 96-well uncoated plates. The cells were exposed for 13 days to either 1 μM paraoxon or 5 μM mipafox beginning at day 1 of differentiation. This time-point (13 days of neurodifferentiation) was chosen as it was considered to be optimal to study cell morphology (based on our own observations). Longer times of neurodifferentiation were not considered appropriate because cell processes were too dense and compromised quantification (data not shown). Cells were washed with PBS buffer and fixed for 15 min at room temperature with 4% paraformaldehyde and then permeabilized for 10 min at room temperature (RT) with 0.1% Triton X-100 in PBS. Cells were incubated for 30 min at RT (room temperature) with blocking buffer (3% FBS in PBS) on a shaker and further incubated overnight at 4 °C with β-Tubulin III (neuron-specific marker) primary antibody (Sigma T8578) diluted 1:200 in blocking buffer. Then, cells were gently washed three times with fresh medium and incubated (1 h at RT in the dark and with horizontal shaking) with fluorophore conjugated with the secondary antibody Alexa 546 (Invitrogen A21123) diluted 1:500 in blocking buffer. At the end of the process cells were incubated for 10 min at RT with 4-6-diamidino-2-phenylindole (DAPI) diluted at 1:5000 in PBS for staining the cell nuclei. DAPI was used for nucleus staining (a fluorescent stain that binds strongly to A-T rich regions in DNA). Cell morphology was analyzed using the imaging platform Cellomics ArrayScan vTi (Thermo Scientific Cellomics^®^, Pittsburgh, PA, USA). The software (Neuronal Profiling version 4, BioApplication from Cellomics Scan Software (Thermo Scientific Cellomics^®^, Pittsburgh, PA, USA)) was set to measure number of branch points, neurite length and percentage of differentiated neurons per total neuron count. The threshold for considering cells as differentiated neurons was more than 3 neurites or a neurite of a total length longer than 6.5 μm associated to a single cell body.

## 5. Conclusions

The obtained results suggest that the NT2 cell line is a suitable *in vitro* model for studying the effect of OPs on the initial stages of RA-induced differentiation of pluripotent stem cells towards neural committed progenitor cells followed up by the early processes of neuronal-like cell differentiation.

Paraoxon and mipafox have been used as examples to study non-neuropathic and neuropathic OPs, respectively. Paraoxon alters the initial *in vitro* differentiation process of neural progenitor cells, probably due to chromosome packaging and chromatin organization. Further studies are needed to clarify whether these effects might cause some of the neurodevelopmental toxic effects attributed to OPs. Nevertheless, we previously estimated that *in vivo* paraoxon exposures causing severe cholinesterase syndrome would yield systemic concentrations of 0.1–0.3 μM [64]. Therefore, 1 μM, the concentration at which the *in vitro* effects reported in this work were observed, would be comparable with clinical cholinergic symptoms. Thus, low risk during developmental stages is expected, which is consistent with data showing alteration in the gene expression profile of D3 mouse embryonic stem cells exposed to chlorpyrifos at concentrations which also cause moderate AChE inhibition [17]. On the other hand, 5 μM of mipafox was able to reduce NTE enzymatic activity. No modifications were observed in mRNA levels, indicating that NTE enzymatic activity is probably not involved in genetic signaling. Our insights into the effects of paraoxon exposure during early windows of cell differentiation call for more data to complete risk characterization since we have studied a single concentration (1 μM) and further concentrations would provide information on where the threshold for the described effects lies.

## Supplementary Information



## Figures and Tables

**Table 1.** Gene set enrichment analysis. Cells were exposed to 1 μM paraoxon for 4 days in RA-induced differentiation medium. All genes with statistically (*p* < 0.05) altered expressions and a fold change higher than 2 or lower than 0.5 were uploaded for further analysis in the DAVID bioinformatics (Nature. Available online: http://www.nature.com/nprot/journal/v4/n1/abs/nprot.2008.211.html (accessed on 26 December 2013).

Function	Number of genes associated with processes altered	Corrected *p*-value
**Cluster Enrichment score = 1.7**		
Chromatin assembly	4	0.001 *
Nucleosome assembly	3	0.010 *
Protein-DNA complex assembly	3	0.011 *
Nucleosome organization	3	0.012 *
Cellular macromolecular complex assembly	4	0.018 *
DNA package	3	0.018 *
Cellular macromolecular complex subunit organization	4	0.024 *
Chromatin organization	4	0.028 *
Chromosome organization	4	0.053 *

**Cluster Enrichment score = 1.33**		
Enzyme binding	6	0.0019 *
Cell death	5	0.036 *
Death	5	0.037 *
Apoptosis	3	0.29
Programmed cell death	3	0.3

**Cluster Enrichment score = 1.04**		
Regulation of cell size	3	0.051 *
Regulation of cellular component size	3	0.083
Neuro differentiation	3	0.18

* *p*-value < 0.05.

**Table 2.** Genes altered in NT2 cells induced by paraoxon during the initial stage of RA-induced differentiation of pluripotent cells towards the neural committed progenitor cells. Cells were exposed to 1 μM paraoxon for 4 days in RA-induced differentiation. Data represent the genes linked to altered mRNA levels compared to control (non-exposed) cultures as identified according to the National Center for Biotechnology Information (NCBI) database.

Gene	Name	NCBI entry	FC real
*HIST1H4E*	histone cluster 1, H4e	8367	−1.69
*LUZP6*	leucine zipper protein 6	767558	−1.57
*LOC400804*	hypothetical LOC400804	400804	−1.24
*HIST1H2AB*	histone cluster 1, H2ab	8335	−1.23
*C14orf162*	chromosome 14 open reading frame 162	56936	−1.19
*YAP1*	Yes-associated protein 1	10413	−1.15
*FGFR1*	fibroblast growth factor receptor 1	2260	−1.05
*AKAP12*	A kinase (PRKA) anchor protein 12	9590	−1.03
*UBE2Z*	ubiquitin-conjugating enzyme E2Z	65264	−1.01
*SRGAP2P2*	SLIT-ROBO Rho GTPase activating protein 2 pseudogene 2	647135	−1.01
*AXIN2*	axin 2	8313	−1.00

*LOC646214*	p21-activated kinase 2 pseudogene	646214	1.00
*LOC100130654*	hypothetical protein LOC100130654	100130654	1.00
*CCR6*	chemokine (C-C motif) receptor 6	1235	1.01
*C14orf135*	chromosome 14 open reading frame 135	64430	1.01
*KIRREL2*	kin of IRRE like 2 (Drosophila)	84063	1.01
*C2orf27A*	chromosome 2 open reading frame 27A	29798	1.02
*BMP8B*	bone morphogenetic protein 8b	656	1.03
*RNF113B*	ring finger protein 113B	140432	1.05
*GCLM*	glutamate-cysteine ligase, modifier subunit	2730	1.06
*TTC16*	tetratricopeptide repeat domain 16	158248	1.07
*PARP4*	poly (ADP-ribose) polymerase family, member 4	143	1.07
*LOC100134868*	hypothetical LOC100134868	100134868	1.07
*SCD5*	stearoyl-CoA desaturase 5	79966	1.08
*PTK2B*	PTK2B protein tyrosine kinase 2 beta	2185	1.08
*LAT2*	linker for activation of T cells family, member 2	7462	1.10
*RPL13AP17*	ribosomal protein L13a pseudogene 17	399670	1.10
*ACBD5*	acyl-CoA binding domain containing 5	91452	1.16
*C1orf152*	profilin 1 pseudogene	767846	1.17
*YY2*	YY2 transcription factor	404281	1.18
*LOC100133791*	hypothetical protein LOC100133791	100133791	1.21
*LOC100233209*	hypothetical LOC100233209	100233209	1.24
*ILDR1*	immunoglobulin-like domain containing receptor 1	286676	1.26
*LOC648740*	actin, beta pseudogene	648740	1.28
*SET*	SET nuclear oncogene	6418	1.29
*LOC100131581*	hypothetical LOC100131581	100131581	1.29
*CXCL5*	chemokine (C-X-C motif) ligand 5 nascent-polypeptide-associated complex alpha polypeptide	6374	1.30
*NACAP1*	pseudogene 1	83955	1.30
*KRTAP10-9*	keratin associated protein 10-9	386676	1.33
*CBX3*	chromobox homolog 3	11335	1.37
*SNRPD2P2*	small nuclear ribonucleoprotein D2 pseudogene 2	645339	1.39
*SGSM2*	small G protein signaling modulator 2	9905	1.42
*ANXA2*	annexin A2	302	1.59
*REREP3*	arginine-glutamic acid dipeptide (RE) repeats pseudogene 3	646396	1.78
*FKSG2*	tumor protein, translationally-controlled 1 pseudogene	59347	1.80
*TXNDC17*	thioredoxin domain containing 17	84817	1.94

## Abbreviations and organophosphorus compounds common names

AChE: Acetylcholinesterase
DAPI: 4-6-diamidino-2-phenylindole
FBS: foetal bovine serum
FGFR1: Fibroblasts growth factor receptor 1
GFAP: Glial fibrillary acidic protein
NT2: human-derived NTera2/D1 cells
MAP2: Microtubule-associated protein 2
Mipafox: *N*,*N*-diisopropyl diamidophosphorofluoridate
MTT: 3-(4,5-dimethylthiazol-2-yl)-2,5-diphenyltetrazolium bromide
NCBI: National Center for Biotechnology Information
NTE: neuropathy target esterase
OPs: organophosphorus compounds
Paraoxon: *O*,*O*′-diethyl *p*-nitrophenyl phosphate
PBS: phosphate buffered saline
PV: phenyl valerate
RA: retinoic acid
SEM: standard error of the mean
YAP1: Yes-associated protein 1
