# Supplementary material for: Genomic and Phenotypic Alterations of the Neuronal-Like Cells Derived from Human Embryonal Carcinoma Stem Cells (NT2) Caused by Exposure to Organophosphorus Compounds Paraoxon and Mipafox
Source: Int J Mol Sci. 2014 Jan 9;15(1):905–26. doi: 10.3390/ijms15010905 (PMC3907846; doi:10.3390/ijms15010905)
Supplement: Supplementary file 1 [file ijms-15-00905-s001.pdf]

Supplementary Information

Figure S1. Additional NTE activity experiment of the hNT2 cells exposed to paraoxon or mipafox during the neurodifferentiation process. This experiment was performed in parallel with the microarray experiment. Cells were exposed to 1 µM paraoxon or 5 µM mipafox for up to 10 days. NTE activity was recorded at the end of each exposure, as described in Section 4.5 and expressed as the percentage regarding activity recorded in the control (non-exposed) cultures. Data represent mean ± SEM of the 8 independent technical replicates for each experimental condition run in a single experiment. A second independent experiment displayed similar results (***** = statistically different from the controls for $p < 0.05$ with a Dunnett's test).

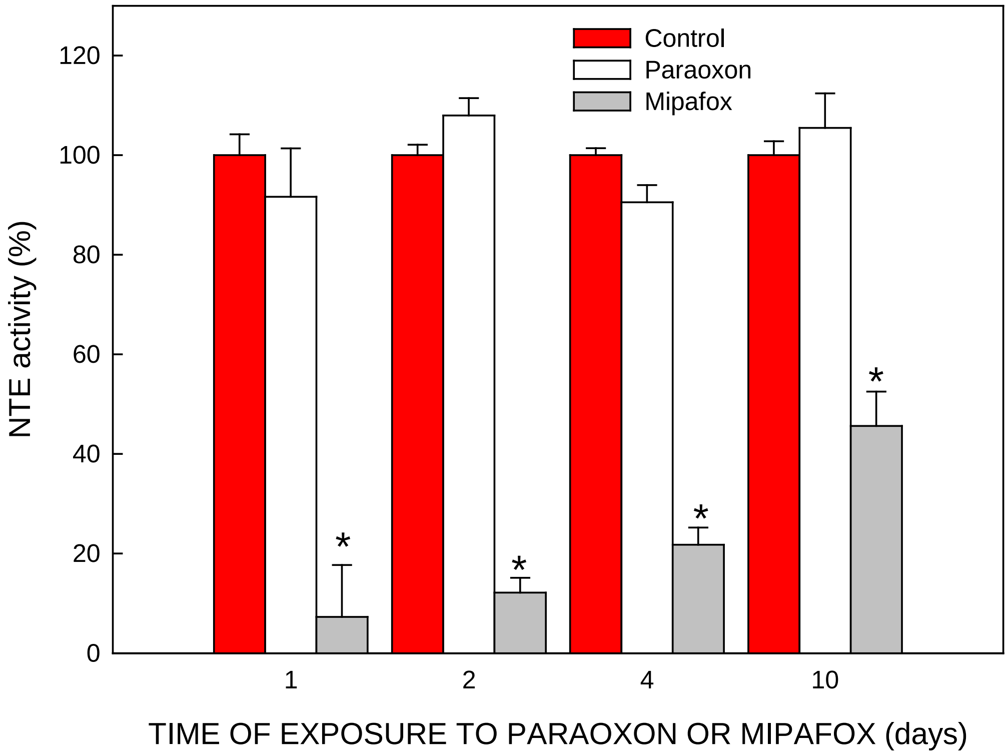

© 2014 by the authors; licensee MDPI, Basel, Switzerland. This article is an open access article distributed under the terms and conditions of the Creative Commons Attribution license (http://creativecommons.org/licenses/by/3.0/).
